# Visual Functions in Patients with Optic Neuritis Pre and Post Treatment: An Observational Study

**DOI:** 10.31729/jnma.9068

**Published:** 2025-06-30

**Authors:** Shailiya Malla, Sabina Shrestha

**Affiliations:** 1 Department of Ophthalmology, Lumbini Eye Institute & Research Center, Siddharthanagar, Rupandehi, Lumbini, Nepal; 2 Department of Ophthalmology, Kathmandu Medical College, Sinamangal, Kathmandu, Nepal

**Keywords:** *Optic neuritis*, *outcome*, *steroid*, *treatment*

## Abstract

**Introduction:**

Optic neuritis is an inflammatory condition affecting the optic nerve. It presents with sudden diminution of vision, also affecting colour vision, contrast sensitivity and stereoacuity. Steroids are given in optic neuritis to reduce inflammation of optic nerve. The aim of this study was to compare the visual functions in patients with optic neuritis before and after treatment with steroid therapy.

**Methods:**

This prospective study was conducted from November 2021 to October 2022 in the Department of Ophthalmology of a tertiary-level hospital. Thirty-seven eyes affected by optic neuritis of were included in the study. All the patients were given IV methylprednisolone for 3 consecutive days followed by oral prednisolone for 11 days in tapering doses. Visual functions such as visual acuity, colour vision, contrast sensitivity and stereoacuity at presentation and after treatment were compared.

**Results:**

Among 37 eyes, 24 (64.86%) had best corrected visual acuity <1/60-perception of light at presentation, improving after six weeks with 21 (56.76%) achieving visual acuity 6/6-6/18. At presentation, only 1 (2.71%) had normal colour vision while at six weeks, 9 (24.32%) had normal colour vision. Contrast sensitivity could not be assessed in 29 (78.37%) cases at presentation due to poor vision, which reduced to 12 (32.43%) at six weeks. None had good stereoacuity at presentation; however, 6 (16.22%) had good stereoacuity after six weeks.

**Conclusions:**

The study showed that steroid therapy improved visual functions in patients with optic neuritis.

## INTRODUCTION

Optic neuritis is an inflammatory condition affecting the optic nerve.^1^ It is termed retrobulbar neuritis when it involves the optic nerve behind the eyeball and optic disc appears normal.^2-4^ It is called papillitis when associated with optic disc swelling.^2-4^ Optic neuritis presents with sudden diminution of vision progressing over several hours to days, also affecting colour vision, contrast sensitivity and stereoacuity.^2^

Steroids are given during treatment of optic neuritis to reduce inflammation of the optic nerve.^5^ Visual recovery is observed to be rapid in patients treated with intravenous methylprednisolone.^5^ In most studies, only visual acuity has been assessed after treatment but colour vison, stereoacuity and contrast sensitivity have not been assessed.^15^

The aim of this study is to compare the visual functions including visual acuity, colour vision, stereoacuity and contrast sensitivity in patients with optic neuritis before and after steroid therapy.

## METHODS

This observational study was conducted from 1st November 2021 to 31st October 2022 in the Department of Ophthalmology of a tertiary-level hospital in Nepal. Data collection was started after obtaining ethical approval from the Institutional Review Committee (IRC) of Kathmandu Medical College [Reference number: 0108202105].

The study included patients with acute clinical presentation consistent with unilateral or bilateral optic neuritis presenting within two weeks of onset of symptoms. Patients with diagnosis of toxic or hereditary optic neuropathy, anterior ischemic optic neuropathy (AION), pregnant women, patients with intracranial neoplasms, and patients taking steroids for other systemic diseases were excluded from the study. Patients with optic atrophy, retinal detachment, and cortical blindness were also excluded from the study.

All patients meeting the inclusion criteria during the study period were enrolled in the study. Informed consent was taken from the eligible patients before recruitment in the study. Detailed history was taken, and examination was done before initiating treatment. All findings were recorded on the proforma.

Visual acuity (VA) for distant vision was assessed by illuminated Snellen vision box with multiple optotypes and E chart (for illiterates) at a distance of six meters. Depending upon the smallest line that the subject could read from 6 m distance, VA was recorded as 6/6, 6/9, 6/12, 6/18, 6/24, 6/36, or 6/60. If VA was < 6/12, the subject was tested with a pinhole to detect the presence of refractive error. If VA was < 6/60, the subject was slowly moved towards the chart until they were able to read the top line in the Snellen vision box, and VA was recorded as 5/60, 4/60, 3/60, 2/60, or 1/60. If the subjects could not read even from 1 m, they were asked to count fingers (CF) shown from a distance of 3, 2, and 1 feet and finger close to face. VA was recorded as CF 3, 2, 1, or CF close to face. If the subject failed to count fingers, the examiner moved their hand close to the subject's face and the subject was asked whether he could appreciate the hand movements or not; VA was recorded as HM+ or HM-. If the subject could not appreciate hand movements, perception of light was noted as PL+ or NPL. Best corrected visual acuity (BCVA) was also assessed by subjective refraction.

Colour vision test was performed using the Ishihara chart. The chart was placed at a distance of 33 cm from the level of patient's eye. Near correction was done in subjects having near vision defect. The patient was asked to identify the numbers shown in the 17 plates. Colour vision was recorded as 1/17, 2/17, 3/17, 4/17, 5/17, 6/17, 7/17, 8/17, 9/17, 10/17, 11/17, 12/17, 13/17, 14/17, 15/17, 16/17, or 17/17 depending upon the number of plates read. If all seventeen plates were read normally, the colour vision was regarded as normal. If thirteen or fewer plates were read normally, colour vision was regarded as deficient. If colour vision could not be assessed due to decreased VA, it was considered absent.

Contrast sensitivity test was performed using Lea contrast flip chart. The charts included the following contrast levels: 25%, 10%, 5%, 2.5%, and 1.25%. From a distance of 3 m, the patient was asked to read the numbers at each contrast level. If the patient was not able to read at 3 m for a given contrast level, the reading distance was reduced to two meters and then to one meter. The lowest contrast level at which the patient was able to read the numbers correctly at a given distance was recorded in percentage. If contrast sensitivity could not be assessed due to decreased VA, it was considered absent.

Stereoacuity was evaluated using Titmus fly test, with polaroid spectacles. Each patient was asked to put on the polaroid spectacles (over his/her own spectacles if present). The Titmus stereoacuity booklet was placed at a distance of 45 cm from the level of patient's eye. The patient was then asked to look at the fly shown and asked what he/she sees, and if he/she can hold the wings of the fly. The examiner noted if the patient picked the wings by touching the page directly or picked the wings in the air. Stereoacuity was recorded accordingly in seconds of arc. Patients were asked to look at the letters under the fly on the right and left side written 'L' and 'R'. If the patient could not read out either 'L' or 'R' then there was either left suppression or right suppression respectively. If suppression was noted, further tests were not carried out. If the patient read out both 'L' and 'R', then no suppression was present and further tests were carried out. On further testing, the patient was shown the circle section of the booklet with nine sets of plates containing four circles in each plate. The patient was asked to choose the circle from each of the nine plates that seemed to be forward when read binocularly. The plate at which the patient was not able to see the circle as more forward was noted in seconds of arc. Near stereoacuity was graded as good (40-60 seconds of arc), moderate (80-200 seconds of arc), poor (> 200 seconds of arc), and absent.

All the patients were given intravenous methylprednisolone 1 gm infusion for three consecutive days followed by oral prednisolone started at 1 mg/kg body weight, which was tapered over 11 days. Ocular examination was again repeated as explained above on day 3, at 2 weeks, and at 6 weeks after initiating treatment. All the findings were recorded on the proforma.

The collected data were checked and entered in Microsoft Excel 2021. The patient's personal information was kept confidential and data secured in a password-protected computer. Data analysis was done using Microsoft Excel 2021 and Epi Info version 7. Non-continuous data were presented as frequency and percentage; continuous data were presented as mean and standard deviation.

## RESULTS

Thirty-seven cases (eyes affected by optic neuritis) in 29 patients were examined and included in the study. Among the 29 patients, optic neuritis was unilateral in 21 (72.41%) and bilateral in 8 (27.59%). The mean age of the patients was 37.00±18.34 years. Among the 37 cases, the etiology was idiopathic in 30 (81.08%). A total of 30 (81.08%) cases were diagnosed with papillitis and 7 (18.92%) were diagnosed with retrobulbar neuritis. The mean duration of presentation was 8.72±4.10 days (Table 1).

**Table 1:** Demographic profile of cases included in the study

Patient Characteristics	n(%)
Total number of patients	29
Total number of cases (eyes affected by optic neuritis)	37
Mean age	37 years (Range 9-75 years)
**Sex**	
Male	16(55.17)
Female	13(44.83)
**Laterality**	
Unilateral	21(72.41)
Bilateral	8(27.59)
**Etiology**	
Idiopathic	30(81.08)
Demyelinating	4(10.81)
Infectious	1(2.71)
Non-infectious	2(5.40)
**Diagnosis (Types of optic neuritis)**	
Papillitis	30(81.08)
Retrobulbar neuritis	7(18.92)
Mean duration	8.72±4.10 days (Range= 1-15 days)

Among the 29 patients, 8 (27.59%) belonged to the 20-29 years age group and 8 (27.59%) to the 30-39 years age group (Table 2).

**Table 2:** Age-wise distribution of patients (n=29)

Age group	n(%)
0-9	1(3.45)
10-19	1(3.45)
20-29	8(27.59)
30-39	8(27.59)
40-49	5(17.24)
50-59	2(6.90)
60-69	1(3.45)
70-79	3(10.33)

Among the 37 cases of optic neuritis, 24 (64.86%) had BCVA <1/60-PL before treatment and at 6 weeks of presentation, 21 (56.76%) had BCVA 6/6 -6/18. (Figure- 1A)

**Figure 1:**
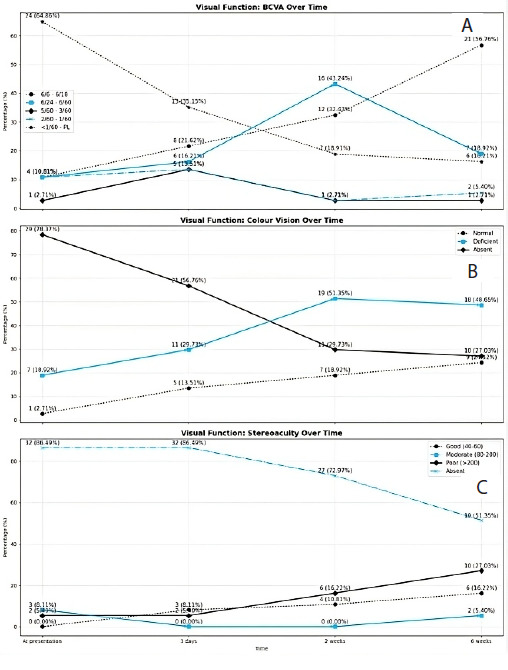
Comparison of visual function outcomes at different time points in cases of optic neuritis. (A) Comparison of best corrected visual acuity (BCVA). (B) Comparison of color vision. (C) Comparison of stereoacuity over time. BCVA=Best corrected visual acuity

Among the 37 cases, colour vision could not be assessed in 29 (78.37%) due to decreased VA at the time of presentation which reduced to 10 (27.03%) cases at 6 weeks. At presentation, only 1 (2.71%) had normal colour vision while at 6 weeks, 9 (24.32%) had normal colour vision. (Figure-1B)

Contrast sensitivity could not be assessed in 29 (78.37%) cases at presentation due to poor vision and was marked as absent, and this reduced to 12 (32.43%) at 6 weeks. All the patients had problems with stereoacuity at the time of presentation. Near stereoacuity was absent in 32 (86.49%) cases before treatment and at 6 weeks of presentation, stereoacuity was absent in 19 (51.35%) cases. None of the cases had good stereoacuity at the time of presentation however at 6 weeks of presentation, 6 (16.22%) cases had good stereoacuity. (Figure-1C)

## DISCUSSION

Optic neuritis usually results in a temporary visual loss.^6^ The first description of optic neuritis by Nettleship in 1884 was described as a failure of sight limited to one eye, often accompanied by pain involving the temple, orbit and with eye movements which usually recover but may be associated with permanent damage and even total blindness.^7^ The Optic Neuritis Treatment Trial (ONTT) was a pioneering study that helped to shape our understanding of optic neuritis and was initially done to evaluate the role of corticosteroids in the management of optic neuritis.^8^ This study also focused on the role of steroids in improvement of visual functions in patients with optic neuritis.

In the present study, the mean age of presentation was 37±18.34 years and majority of the patient's age group was 20-29 years and 30-39 years. It is widely accepted that optic neuritis usually affects young adults.^7^ Since majority of the patients belonged to age group of 20-29 and 30-39 years in the present study, the findings of our study support this. The mean age of the patients is also comparable to previous studies.^8-12^ The mean duration of presentation was 8.72±4.1 days. Similar result was seen in other studies.^9-10^

Males and females comprised of 16 (55.17%) and 13 (44.83%) respectively with male to female ratio of 1.23:1 in our study. Similarly, higher male preponderance was found in other studies by Rajkarnikar et al., Das et al., and Shrestha et al.^9,10,13^ In contrast, female predominance was found in ONTT.^14^ Also, studies done by Godar et al. and Thapa BB and Dhakal Y showed female predominance.^11-12^

Optic neuritis usually presents as an acute, unilateral or bilateral vision loss which is usually painful.^15^ Among 29 patients, unilateral disease was seen in 21 (72.41%) patients, and bilateral disease was seen in 8 (27.59%) patients. Similar results were found in most of the previous studies.^8-9,11^ However, a study conducted in Eastern Nepal showed higher number of cases of bilateral optic neuritis.^10^ Another study from central Nepal found equal number of unilateral and bilateral cases.^13^

In this study, papillitis was more common than retrobulbar neuritis which was similar to other studies from Nepal.^11,13^ A study conducted in India also showed similar results.^8^ However, ONTT showed retrobulbar neuritis as the predominant type of optic neuritis.^14^ Similarly, in the study done by Rajkarnikar et al., retrobulbar optic neuritis was more common.^9^

In the present study, idiopathic aetiology was found to be most common cause of optic neuritis accounting for 30 (81.08%) cases. The commonest cause of optic neuritis was found to be idiopathic in other studies as well.^11,13,15-16^ Idiopathic optic neuritis was the most common cause in all age groups in our study. However, in the study done in Thailand, neuromyelitis optica spectrum disorder was common in younger patients as compared to multiple sclerosis and idiopathic group.^17^ Among patients older than 45 years, no aetiology other than idiopathic optic neuritis was found in our study. However, in the study done by Hudzaifah-Nordin et al., although most cases were idiopathic optic neuritis, few other aetiologies like infectious, neuromyelitis optica spectrum disorder were also found to be the cause of optic neuritis in patients older than 45 years.^18^

There was one case of multiple sclerosis and three cases showed clinical signs of neuromyelitis optica spectrum disorder. Both of these demyelinating diseases were diagnosed among females in our study. This finding was consistent with the study done in Thailand where neuromyelitis optica spectrum disorder and multiple sclerosis were seen commonly among females.^17^ Very few cases of optic neuritis due to autoimmune cause was seen in our study which was similar to the study done by Ismail et al.^16^ There was only one case of optic neuritis due to infectious cause (due to syphilis) in our study which was similar to the study done by Wang et al.^19^

The most common symptom was diminution of vision which was found in all patients. Other common symptoms were headache and painful eye movements in present study. Visual acuity in the affected eye was poor at the time of presentation in most of our patients. None of our patients had vision of NPL. Likewise in the study done by Rajkarnikar et al., there were no patients with NPL.^9^ However, most other studies included patients presenting with NPL during presentation.^10-12,14^ We believe that our patients presented early for ophthalmology consultation due to diminution of vision and received treatment before the vision dropped to NPL. Due to easy access to health care in Kathmandu valley, people nowadays are able to receive timely treatment. This might be the reason that in this study there were no patients with VA of NPL.

All the patients in our study were treated according to the same guidelines as used in the Optic Neuritis Treatment Trial.^15^ In the present study, 64.86% cases had BCVA <1/60-PL before treatment and at 6 weeks, 56.76% cases had BCVA 6/6 -6/18. The improvement in BCVA after treatment was found to be statistically significant. Almost all of the eyes responded well to treatment, which is in accordance with the ONTT and other studies in Nepal.^10,12,20,21^

All the patients in our study were treated according to the same guidelines as used in the Optic Neuritis Treatment Trial.^15^ In the present study, 24 (64.86%) cases had BCVA <1/60-PL before treatment and at 6 weeks, 21 (56.76%) cases had BCVA 6/6-6/18. Almost all of the eyes responded well to treatment, which is in accordance with the ONTT and other studies in Nepal.^10,12,20-21^

Colour vision could not be assessed in 29 (78.37%) cases due to decreased visual acuity at the time of presentation and was noted as absent. Colour information and other static elements of visual perception, is carried through the parvocellular fibres in the central region of the optic nerve. Therefore, diseases of the optic nerve have been thought to affect colour vision even in the presence of good visual acuity.^22^ Moreover, an increased vulnerability of the parvocellular system has been documented to relatively selective injury due to their smaller diameter making them either more susceptible to demyelination or less capable of repair.^22^ The percentage of patients with absent colour vision reduced to 21 (56.76%) after 3 days of intravenous methylprednisolone. This further reduced to 11 (29.73%) at 2 weeks, after treatment with 11 days of oral Prednisolone. At 6 weeks, colour vision could not be assessed in only 10 (27.03%) of cases. 9 (24.32%) of the cases reverted back to normal colour vision at 6 weeks. This finding was similar to the study done by Shrestha et al.^13^

Contrast sensitivity was decreased in all the patients at the time of presentation which was similar to other studies.^11,23-24^ Contrast sensitivity could not be assessed in 78.37% cases at presentation due to poor vision and was marked as absent and this reduced to 32.43% at 6 weeks. At 6 weeks follow- up, majority of eyes 40.60% had contrast sensitivity of 1.25% at one meter distance. Improvement of contrast sensitivity was also noted in the study done by Saxena et al.^8^ Although improvement of contrast sensitivity was seen, none of the patients had normal contrast sensitivity at last follow up in this study. Therefore, many patients after recovering from optic neuritis complained of vision being 'washed out' or 'dull' despite visual acuity being 6/6. Decrease in contrast sensitivity in optic neuritis may result from the demyelinating damage to the optic nerve, which may be either selective and include mainly small axons, or generalized including both small and large axons.^25^

Near stereoacuity was also assessed in the present study. All of the patients had problems with stereoacuity at the time of presentation. Near stereoacuity was absent in 32 (86.49%) cases before treatment. At 6 weeks of presentation, stereoacuity was absent in 19 (51.35%) cases which indicated improvement after treatment. No cases had good stereoacuity at the time of presentation however at 6 weeks of presentation, 6 (16.22%) cases had good stereoacuity. We found no other studies assessing the stereoacuity in patients with optic neuritis.

Since steroids has anti-inflammatory properties, it helps reduce inflammation of optic nerve which may aid in improvement of vision.^26^

There are a few limitations of our study. Firstly, we only followed up the cases for up to 6 weeks, so further improvement after 6 weeks was not assessed. In addition, we did not study any complications that occurred in our patients due to treatment with steroids. We suggest further studies with long-term follow up and also assessing the complications occurring from treatment. Another limitation was that contrast sensitivity was taken using Lea contrast flip chart instead of Peli Robertson contrast sensitivity chart, due to unavailability of this chart, which would have given better recordings. Furthermore, we did not have a control group in whom steroid treatment was not given since it would have been unethical to not give treatment to the patients.

## CONCLUSION

Patients diagnosed with optic neuritis showed improvement in visual functions including visual acuity, colour vision, contrast sensitivity and stereoacuity after treatment with steroids.
